# Plakophilin 1 in cancer: context-dependent dualism, subcellular dynamics, and therapeutic targeting

**DOI:** 10.3389/fcell.2025.1703626

**Published:** 2025-12-11

**Authors:** Yu-Mei Huang, Wen-Ling Zhang, Fa-Qing Tang

**Affiliations:** 1 Clinical Laboratory of Hunan Cancer Hospital, The Affiliated Cancer Hospital of Xiangya School of Medicine, Central South University, Hunan Key Laboratory of Oncotarget Gene, Changsha, Hunan, China; 2 Department of Clinical Laboratory, The Third Xiangya Hospital, Central South University, Changsha, Hunan, China

**Keywords:** biomarker, desmosomal plaques, mRNA translation, Plakophilin 1, therapeutic target, tumorigenesis

## Abstract

Plakophilin 1 (PKP1) is a desmosomal protein that plays a dual role in cancer, acting as either an oncogene or a tumor suppressor depending on the context. This review consolidates evidence regarding its mechanistic regulation via crucial signaling pathways, including Wnt/β-catenin, PI3K/AKT, and MAPK and underscores its potential as a clinical biomarker. The function of PKP1 is influenced by its subcellular localization: when membrane-bound, PKP1 stabilizes cell adhesion, whereas in the cytoplasm or nucleus, it facilitates oncogenesis by promoting MYC translation and modulating DNA damage. Phosphorylation events further refine this functional plasticity. Clinically, PKP1 expression is valuable for cancer subtyping and prognosis. This review also addresses unresolved questions concerning its regulation and suggests that future research in these areas could unlock the potential of PKP1 in precision oncology strategies.

## 1 Introduction

Plakophilin 1 (PKP1), an 82 kDa armadillo-repeat protein and a core component of desmosomal plaques, is essential for maintaining intercellular adhesion and epithelial integrity through well-documented mechanisms (Green and Gaudry, 2000; Fuchs et al., 2019; Bass-Zubek et al., 2009; Lee and McGrath, 2021). Desmosomes, as specialized intercellular junctions, provide mechanical resilience to tissues under high stress while dynamically regulating cellular motility (Green and Gaudry, 2000; Fuchs et al., 2019; Bass-Zubek et al., 2009; Lee and McGrath, 2021). This dual functionality has significant implications for cancer pathogenesis. Experimental studies have shown that the depletion of PKP1 in keratinocytes leads to destabilization of desmosomal architecture and an increase in migratory capacity, mimicking the epithelial-mesenchymal transition (EMT) characteristics observed in invasive carcinomas (South et al., 2003). Collectively, these findings highlight PKP1 as a crucial modulator in tumor biology. In human malignancies, PKP1 demonstrates a context-dependent functional dichotomy, challenging the traditional classification of oncogenes and tumor suppressors. This review employs a multi-tiered approach to address existing knowledge gaps: firstly, it systematically compares the oncogenic and tumor-suppressive roles of PKP1 across various cancer types to elucidate tissue-specific mechanisms. secondly, it incorporates emerging evidence on post-translational regulation and subcellular trafficking to characterize context-dependent functional switches. finally, it assesses the translational relevance of PKP1 by examining its diagnostic, prognostic, and therapeutic targeting potential across various oncological settings.

## 2 The context-dependent dual roles of PKP1 in cancer

PKP1 expression is subject to dynamic regulation during oncogenesis and tumor progression, exhibiting strong correlations with tumor differentiation, aggressiveness, and metastatic potential. Analyses utilizing the Oncomine™ platform reveal significant dysregulation of PKP1 across a range of malignancies, with particularly notable alterations in certain cancer types (Hofmann, 2020). As outlined in Table 1, PKP1 displays a paradoxical, context-dependent duality, acting as a tumor suppressor in some cancers while serving as an oncogene in others.

**TABLE 1:** Context-dependent roles of PKP1 across different cancer types.

Cancer type	Expression pattern	Subcellular localization	Proposed role	Key mechanisms	Clinical correlation	Refs
NSCLC, Squamous	Upregulated	Cytoplasm/Nucleus (Oncogenic); Membrane (diagnostic)	Oncogene and Diagnostic Marker	Enhance MYC translation; Stabilizes PFKP to Drive Glycolysis; Stabilizes desmosomes	A diagnostic biomarker and potential therapeutic target	Martin-Padron et al. (2020), Boyero et al. (2022), Ritoré-Salazar et al., 2025.; Gómez-Morales et al. (2013), Galindo et al. (2020), Sanchez-Palencia et al. (2011), Hatzfeld (2007)
NSCLC, Adenocarcinoma	Downregulated	Cytoplasmic/Nuclear (no membrane staining)	Diagnostic Marker (Negative)	Lack of adhesive function due to loss of membrane localization	Used to rule out SCC in diagnostic panels Part of multi-marker assays	Gómez-Morales et al. (2013), Galindo et al. (2020)
NPC	Upregulated	Cytoplasmic	Oncogene	Immunosuppression via MDSCs; Impairs B-cell proliferation	Correlates with reduced progression-free survival (PFS)	Huang et al. (2022)
EAC	Downregulated	Loss from Membrane	Tumor Suppressor	Promoter hypermethylation; Desmosome destabilization	Accelerates progression from Barrett’s esophagus	Kaz et al. (2012)
Esophageal SCC	Downregulated	Loss from Membrane	Tumor Suppressor	Loss of expression and phosphorylation	Low expression predicts poorer overall survival; Independent prognostic factor in multi-gene models	Zhang et al. (2025a)
Oropharyngeal SCC	Downregulated	Lost in Metastasis; Cytoplasmic in recurrence	Tumor; Suppressor	Loss of adhesion	Correlates with tumor dedifferentiation and poor prognosis; Undetectable in metastatic tumors	Papagerakis et al. (2003)
PCad	Downregulated	Loss from Membrane	Tumor Suppressor	Prolongs stability of proinflammatory cytokine mRNAs (IL-6, IL-8, CXCL1)	Associated with aggressive phenotypes (Gleason 8–10) and lymph node metastasis; Creates immunosuppressive TME	Breuninger et al. (2010), Yang et al. (2013), Kim et al. (2023)
Cervical cancer	Downregulated	nucleus	Tumor Suppressor	Loss of adhesion	Progressively declines from normal epithelium to invasive SCC	Schmitt-Graeff et al. (2007)
Cutaneous SCC	Downregulated	Loss from Membrane	Tumor Suppressor	Phosphorylation of PKP1 by RIPK4	Significantly higher in well-differentiated vs. poorly differentiated tumors	Moll et al. (1997), Schwarz et al. (2006), Lee et al. (2017)
Breast Cancer	Upregulated	Cytoplasmic	Oncogene	Phosphorylation of PKP1 by AKT2; LncRNA APPAT/miR-328a axis	Correlates with decreased overall survival (OS) and disease progression	Li et al. (2021), Wang et al. (2020), Wolf et al. (2013)

NSCLC: Non-small cell lung cancer; NPC: nasopharyngeal carcinoma; EAC: esophageal adenocarcinoma; SCC: squamous cell carcinoma; PCad: Prostate adenocarcinoma; PFKP: platelet-type phosphofructokinase; CTCs: Circulating Tumor Cells; TME: tumor microenvironment.

In the squamous cell carcinoma (SCC) subtype of non-small cell lung cancer (NSCLC), PKP1 exhibits dual roles: cytoplasmic/nuclear localization promotes tumor progression via MYC translation and metabolic reprogramming, while membrane retention serves as a diagnostic marker for SCC subtyping (Martin-Padron et al., 2020; Boyero et al., 2022; Ritoré-Salazar et al., 2025; Gómez-Morales et al., 2013; Galindo et al., 2020). This spatial segregation of functions within the same cancer type highlights PKP1’s complex regulatory landscape.

Accumulating evidence suggests that PKP1 functions as a tumor suppressor in various cancer contexts. This role is exemplified in cervical carcinogenesis, where PKP1 expression progressively diminishes from normal epithelium (NE) to low-grade cervical squamous intraepithelial lesions (SILs), high-grade SILs, and ultimately invasive SCC (Galindo et al., 2020). This pattern of progressive loss during disease progression strongly supports its tumor-suppressive function. Similarly, oropharyngeal SCC is characterized by significantly reduced levels of PKP1 compared to NE cells (Papagerakis et al., 2003). This finding is further substantiated in prostate and esophageal cancers. In prostate adenocarcinoma (PCad), the downregulation of PKP1 in aggressive phenotypes is associated with enhanced proinflammatory cytokine stability, thereby fostering an immunosuppressive microenvironment that accelerates cancer progression (Breuninger et al., 2010; Yang et al., 2013; Kim et al., 2023). Similarly, during the progression from Barrett’s esophagus to adenocarcinoma, hypermethylation of the PKP1 promoter results in its silencing, which contributes to desmosome destabilization and tumorigenesis (Kaz et al., 2012). In esophageal SCC, PKP1 is a vital component of a four-gene prognostic signature (CCND1-PKP1-JUP-ANKRD12), where its low expression is independently predictive of poorer overall survival (Zhang X. et al., 2025).

Conversely, PKP1 exhibits oncogenic properties in nasopharyngeal and breast cancers. In nasopharyngeal carcinoma (NPC), overexpression of PKP1 is associated with reduced progression-free survival, as it impairs B-cell proliferation through pathways mediated by myeloid-derived suppressor cells (MDSC) (Huang et al., 2022). In breast cancer models, PKP1 facilitates the formation of circulating tumor cell (CTC) cluster via activation of the PI3K/AKT/Bcl-2 pathway, thereby enhancing metastatic survival in the bloodstream (Li et al., 2021). These findings identify PKP1 as a multifaceted oncoprotein in epithelial malignancies.

Clinically, the expression patterns of PKP1 exhibit a strong correlation with differentiation status and metastatic potential. In cutaneous and oropharyngeal SCCs, the retention of PKP1 is associated with well-differentiated histology and a lower risk of metastasis, whereas its loss is indicative of dedifferentiation and poor prognosis (Papagerakis et al., 2003; Moll et al., 1997; Schwarz et al., 2006). Notably, PKP1 becomes undetectable in metastatic oropharyngeal SCC, while nonmetastatic tumors maintain high levels of expression, emphasizing its role in suppressing metastasis (Papagerakis et al., 2003).

## 3 Molecular mechanisms of PKP1 dualism

The context-dependent duality of PKP1—acting as either a tumor suppressor or an oncoprotein—raises a fundamental question in cancer biology: what molecular mechanisms govern these opposing functions? Emerging evidence suggests that the functional output of PKP1 is not dictated by a single pathway but is instead governed by a dynamic interplay of spatially and temporally regulated processes.

### 3.1 Dynamic regulation of localization and post-translational modifications

#### 3.1.1 Subcellular Localization

PKP1 serves as a multifunctional scaffold protein, with its subcellular localization and post-translational modifications playing a critical role in modulating cellular behavior under both physiological and neoplastic conditions. The distinct subcellular distribution of PKP1 is associated with opposing functional outcomes: membrane-bound PKP1 suppresses tumorigenesis, whereas cytoplasmic and nuclear PKP1 promote oncogenesis.

In normal esophageal squamous cells, PKP1 is localized to desmosomal plaques through interactions with desmogleins, desmocollins, and desmoplakin (Lee and McGrath, 2021; Kaz et al., 2012). This membrane-bound form of PKP1 enhances intercellular adhesion, suppresses EMT, and restricts cellular proliferation. Notably, the armadillo repeats domain (ARD) of PKP1 colocalizes with actin within lamellipodia, facilitating the formation of filopodia and promoting directed cell migration (Hatzfeld et al., 2000).

##### 3.1.1.1 Cytoplasmic PKP1: oncogenic translation machinery

The loss of membrane-associated PKP1 is a characteristic feature of carcinogenesis, as observed in precancerous Barrett’s esophagus and EAC (Hatzfeld, 2007). In oropharyngeal SCC, although PKP1 is present in both membrane and cytoplasmic compartments, locally recurrent tumors predominantly exhibit cytoplasmic localization (Papagerakis et al., 2003). This aberrant redistribution enables PKP1 to exert its oncogenic roles: within the cytoplasm, PKP1 nucleates mRNA regulatory complexes, notably within the stress granules (Santofimia-Castano et al., 2021), and interacts with the DEAD-box RNA helicase eIF4A1 to enhance the translation of oncogenic mRNAs, such as MYC (Wolf et al., 2010).

Additionally, cytoplasmic PKP1 further assembles with PKP3 and various RNA-binding proteins (e.g., FXR1, G3BP, PABPC1, and UPF1) to form messenger ribonucleoprotein particles that critically regulate the stability and translational efficiency of target transcripts (Fischer-Kešo et al., 2014). Through these interactions, PKP1 directly binds to the 5′-UTR of MYC and, through FXR1, stabilizes MYC mRNA via its AU-rich 3′-UTR, thereby establishing a potent mechanism for promoting oncogenesis (Martin-Padron et al., 2020; Boyero et al., 2022). At the post-transcriptional level, miR-328a mitigates the oncogenic effects of PKP1 by directly targeting it (Wang et al., 2020), while the lncRNA APPAT acts as a miR-328a sponge to modulate PKP1 expression in breast cancer (Wang et al., 2020).

##### 3.1.1.2 Nuclear PKP1: transcriptional and epigenetic modulator

Nuclear PKP1 functions as a transcriptional and epigenetic regulator, facilitated by a nuclear localization sequence within its N-terminal domain (amino acids 56–125) (Schmidt et al., 1997; Schmidt and Jäger, 2005; Sobolik-Delmaire et al., 2010). It establishes a positive feedback loop with MYC(7, 8) and interacts with essential nuclear regulators. Specifically, PKP1 associates with nuclear protein 1 (NUPR1), a transcriptional regulator involved in DNA damage responses and chromatin remodeling (Santofimia-Castano et al., 2021). The PKP1/NUPR1/PADI4 complex catalyzes histone citrullination, leading to the repression of p53 target genes and the promotion of DNA fragmentation (Neira et al., 2023; Araujo-Abad et al., 2023; Yang et al., 2021; Wang et al., 2021; Tanikawa et al., 2012). Notably, p53 transactivates PADI4, which reciprocally represses p53 (35). DNA damage triggers the redistribution of PKP1 to the nucleoli, and its depletion under these conditions enhances cell survival (Sobolik-Delmaire et al., 2010). To counteract its pro-oncogenic effects, PKP1 interacts with RYBP (a component of PRC1) in both the nucleus and cytoplasm, an interaction that mitigates vimentin-driven EMT (Araujo-Abad et al., 2024).

##### 3.1.1.3 Phosphorylation

Posttranslational modifications, especially phosphorylation, are crucial in determining the subcellular localization and function of PKP1. In the absence of growth factor signaling, unphosphorylated PKP1 forms stable complexes with desmoplakin, thereby facilitating desmosome assembly and cell‒cell adhesion (Wolf et al., 2013). Under these conditions, excess cytoplasmic PKP1 undergoes targeted degradation. Conversely, stimulation by growth factor results in AKT2-mediated phosphorylation at Ser118, which inhibits PKP1 degradation and consequently leads to its accumulation in the cytoplasm (Wolf et al., 2013). Cytoplasmic PKP1 exhibits oncogenic properties by promoting desmosomal remodeling through weakened interactions, enhancing cap-dependent mRNA translation (resulting in an approximately 250% increase in MYC translation), and driving cell proliferation (Wolf et al., 2013). However, phosphorylation at Ser143 by RIPK4, located within the N-terminal domain of PKP1, promotes epidermal differentiation and maintains its tumor-suppressive function, underscoring the delicate balance regulated by kinase activities (Lee et al., 2017).

##### 3.1.1.4 Metabolic reprogramming

Metabolic reprogramming is a hallmark of human cancer, offering opportunities for cancer diagnosis, prognosis, and treatment (Lin et al., 2024). PKP1 also plays a direct role in reprogramming cancer cell metabolism. A seminal study unveiled a novel oncogenic mechanism by which PKP1 drives a hypermetabolic state in SCC of NSCLC (9). Through a genome-wide CRISPR screen, it is identified that cells expressing PKP1 exhibit a critical dependence on mitochondrial function and glycolysis. Mechanistically, PKP1 interacts with the E3 ubiquitin ligase TRIM21, preventing it from ubiquitinating and degrading platelet-type phosphofructokinase (PFKP), a pivotal rate-limiting enzyme in glycolysis. By stabilizing PFKP, PKP1 enhances glycolytic flux, thereby promoting tumor proliferation (Ritoré-Salazar et al., 2025). Additionally, PFKP has been demonstrated to augment the ERK-mediated stability of the c-Myc oncoprotein, establishing a direct connection between the metabolic role of PKP1 and the enhancement of a central oncogenic driver (Liu et al., 2024). This PKP1-PFKP-MYC axis constitutes a direct link between a desmosomal protein and metabolic reprogramming, presenting a novel therapeutic target for NSCLC.

**FIGURE 1:**
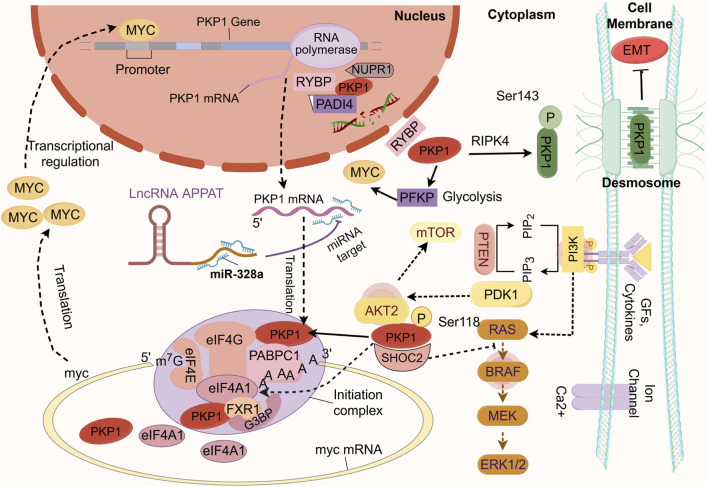
Schematic overview of PKP1’s dual functions in cancer. Tumor-suppressive role (green theme, at the cell membrane): Membrane-localized PKP1 is a core component of desmosomes, where it stabilizes cell-cell adhesion and inhibits EMT, thereby suppressing tumor invasion and metastasis. Oncogenic role (red theme, in the cytoplasm and nucleus): Upon loss of membrane localization, PKP1 exerts oncogenic functions. In the cytoplasm, PKP1 can be phosphorylated by AKT2 at Ser118. Phosphorylated PKP1 binds to the RNA helicase eIF4A1, facilitating the assembly of a translation initiation complex that enhances the synthesis of the MYC oncoprotein, driving proliferation and invasion. Meanwhile, PKP1 stabilizes key glycolytic enzymes like PFKP, enhancing glycolytic flux (Warburg effect) to fuel cancer cell energy metabolism and biomass synthesis. In the nucleus, PKP1 forms a complex with NUPR1 and PADI4, which catalyzes histone citrullination, modulates gene transcription and the DNA damage response. A positive feedback loop with MYC further amplifies its oncogenic potential. The subcellular localization and post-translational modifications of PKP1 serve as a critical switch between its opposing functions in tumorigenesis. AKT2: AKT serine/threonine kinase 2; eIF4A1: eukaryotic translation initiation factor 4A1; EMT: epithelial-mesenchymal transition; GFs: growth factors; MYC: MYC proto-oncogene; NUPR1: nuclear protein 1; PADI4: peptidyl arginine deiminase 4; PKP1: plakophilin 1. Created by Figdraw.

In conclusion, the biological functions of PKP1 are significantly influenced by a dynamic interplay between its subcellular localization and specific protein interactions, which underpin its context-dependent dualism in cancer. The prevailing model suggests a spatial functional switch: Membrane-bound PKP1 maintains epithelial integrity by stabilizing desmosomal adhesion, thereby exerting a tumor-suppressive role. Conversely, aberrant localization to the cytoplasm or nucleus endows PKP1 with oncogenic potential: cytoplasmic PKP1 drives oncoprotein synthesis by enhancing mRNA translation (e.g., MYC) and reprograms cellular metabolism by stabilizing glycolytic enzymes such as PFKP, while nuclear PKP1 modulates transcription and DNA damage responses. This spatial and functional segregation, further refined by phosphorylation, ultimately dictates whether PKP1 constrains or promotes tumor progression (Figure 1). Although this model is well-supported by correlative data, future research using compartment-specific targeting is needed to confirm a direct link between the location of PKP1 and its effects.

### 3.2 Genetic alterations and epigenetic changes

Germline mutations in the PKP1 gene, including homozygous splice-site variants (1233-2 A→T and 2021 + 1 G>A) and heterozygous acceptor site mutations, are implicated in ectodermal dysplasia–skin fragility syndrome (McGrath et al., 1997; Whittock et al., 2000; Hamada et al., 2002; McMillan et al., 2003). These splicing defects result in PKP1 deficiency, manifesting as skin fragility and developmental abnormalities (McGrath et al., 1997). Functionally, the loss of PKP1 contributes to the creation of a pro-oncogenic microenvironment, supporting its role as a tumor suppressor. Concurrently, epigenetic silencing via promoter methylation represents a key mechanism underlying PKP1 dysregulation in cancer. For instance, Barrett’s esophagus and EAC exhibit hypermethylated PKP1 promoters with concomitant expression loss (Kaz et al., 2012). Similarly, lung cancer cell lines exhibit PKP1 downregulation associated with promoter methylation (Haase et al., 2019), emphasizing the recurrent nature of epigenetic regulation in carcinogenesis.

### 3.3 Signal pathway integration

The functional duality of PKP1 is mediated through its integration into critical oncogenic signaling pathways, including Wnt/β-catenin, Ca^2+^, PI3K/AKT, and MAPK, which collectively influence cell fate decisions in cancer.

#### 3.3.1 Wnt/β-catenin pathway and Ca^2+^ signaling

As an armadillo repeat protein, PKP1 shares structural homology with β-catenin, suggesting its potential role as an effector in Wnt signal transduction (Choi and Weis, 2005). During tooth development, Wnt signaling facilitates the translocation of PKP1 from the membrane to the nucleus via its N-terminal domain (amino acids 161–270), thereby establishing its role as a downstream component of the canonical Wnt cascade (Miyazaki et al., 2016). Mechanistically, PKP1 interacts with the tight junction protein ZO-1, modulating its distribution and regulating ameloblast differentiation (Miyazaki et al., 2016).

Additionally, PKP1 mediates a critical transition in desmosomal adhesion, shifting from calcium-dependent to calcium-independent states (Tucker et al., 2014). This characteristic enables PKP1 to protect keratinocytes from IgG-induced adhesion disruption in aspergillosis (Tucker et al., 2014). In metastatic melanoma, overexpression of PKP1 is associated with enriched calcium signaling pathways (Wang et al., 2019). Extracellular Ca^2+^ strongly induces PKP1 expression (Miyazaki et al., 2016), triggering its nuclear-to-membrane translocation where it colocalizes with β-catenin to stabilize adherent junctions. Conversely, stimulation with Wnt3a or inhibition of GSK3β (via LiCl) promotes the shuttling of PKP1 from the membrane to the nucleus, resulting in the dissociation of β-catenin from junctions and facilitating its nuclear translocation (Miyazaki et al., 2016). The reciprocal regulation between Ca^2+^ and Wnt signaling creates a PKP1-mediated switch that toggles between “adhesion maintenance” (high Ca^2+^) and “proliferation/migration” (Wnt activation), with significant implications for cancer metastasis and tissue homeostasis (Miyazaki et al., 2016).

#### 3.3.2 PI3K/AKT pathway

Additionally, the PI3K/AKT cascade represents another crucial oncogenic pathway modulated by PKP1 (He et al., 2021). Among the AKT isoforms, AKT2 is notably associated with tumor metastasis and chemotherapy resistance (Revathidevi and Munirajan, 2019). PKP1 interacts specifically with AKT2 in an activity-dependent manner (Wolf et al., 2013), demonstrating dual roles that are context-dependent: in the presence of insulin/IGF1, PI3K/AKT2 signaling phosphorylates PKP1 at Ser118, leading to its accumulation in the cytoplasm. This phosphorylation event enhances eIF4A-mediated mRNA translation, weakens intercellular adhesion, and promotes cellular proliferation and invasion (Wolf et al., 2013). In contrast, unphosphorylated PKP1 localizes to desmosomes, where it stabilizes junctions and suppresses tumorigenesis (Wolf et al., 2013). In breast and lung cancer, overexpression of PKP1/DSC2 activates the PI3K/AKT/Bcl-2 pathway, promoting CTC cluster formation. These clusters resist shear stress-induced apoptosis, thereby enhancing metastatic survival (Li et al., 2021). Consequently, the AKT-PKP1 axis serves as a pivotal switch between adhesion and proliferation.

#### 3.3.3 MAPK signaling

PKP1 also exerts context-dependent effects on MAPK pathways. Receptor-interacting serine/threonine-protein kinase 4 (RIPK4) phosphorylates PKP1 at Ser143, facilitating the formation of the PKP1-SHOC2 complex. This interaction inhibits Ras/MAPK signaling, leading to the upregulation of differentiation markers (Krt10, loricrin), while concurrently suppressing cellular proliferation (Lee et al., 2017; Fortugno et al., 2022; Kwon et al., 2022). Keratinocytes deficient in PKP1 display impaired differentiation and exhibit invasive cutaneous SCC phenotypes (Moll et al., 1997). In contrast, within CTC clusters, the PKP1/DSC2 complex anchors vimentin to form hybrid epithelial-mesenchymal complexes. This configuration activates the ITGB1-FAK-Src axis, thereby stimulating MEK-ERK signaling and enhancing metastatic survival (Li et al., 2021). Notably, knockdown of PKP1 alone does not significantly reduce ERK1/2 phosphorylation, indicating the presence of compensatory mechanisms (Li et al., 2021). Therefore, the involvement of PKP1 in MAPK signaling is context-dependent, either suppressing the pathway through RIPK4 to promote differentiation or activating it in CTCs to facilitate metastasis.

During carcinogenesis, PKP1 dynamically modulates malignant transformation, invasion, and migration through its interactions with MYC, RAS, RNA-binding proteins, eukaryotic initiation factors (eIFs), and noncoding RNAs. This establishes a complex regulatory network in which PKP1 acts as a central hub, bridging adhesion dynamics, translation control, and signal transduction (Figure 1). The functional roles of PKP1 in tumor suppression versus progression are critically influenced by its subcellular localization, phosphorylation status, and protein interactions.

### 3.4 Modulation of the tumor microenvironment

PKP1 has been identified as a pivotal regulator of the TME, with its influence being both profound and context-dependent. In PCad, a deficiency in PKP1 is characteristic of aggressive phenotypes and is directly linked to a significant increase in the infiltration of diverse immune cells, including T cells, B cells, macrophages, and neutrophils, into tumor areas (Kim et al., 2023). This process is driven by PKP1’s canonical function as an RNA-binding protein. PKP1 directly binds to and destabilizes the mRNAs of key proinflammatory cytokines (CXCL1, IL-6, IL-8). Consequently, the knockdown of PKP1 results in a substantial increase in the stability and half-life of these cytokine mRNAs, leading to their accumulation and subsequent elevation in protein secretion (Kim et al., 2023). This process directly fosters an immunosuppressive niche that recruits THP-1 cells and peripheral blood mononuclear cells to accelerate progression and metastasis (Kim et al., 2023).

However, the immunomodulatory narrative of PKP1 is not monolithic. In NPC, PKP1-positive tumor cells exert immunosuppressive effects through a distinct mechanism. These cells impair B-cell proliferation via MDSC-mediated induction of inducible nitric oxide synthase and NADPH oxidase 2 (He et al., 2021). This pathway correlates with reduced tumor-infiltrating B cells and worse clinical outcomes (Huang et al., 2022). This highlights the critical importance of cellular context and tumor type in the immunological effects of PKP1, ranging from pro-inflammatory (in PCad loss) to immunosuppressive (in NPC gain).

To comprehensively elucidate the causality and compartmental specificity of these effects, future research must employ advanced genetic models. Employing cell-type-specific conditional knockout strategies, such as selectively deleting *PKP1* in epithelial cells versus specific immune lineages will be essential. These methodologies can definitively ascertain whether the immunomodulatory functions of PKP1 are inherent to the tumor cell or necessitate its activity within immune populations. Furthermore, integrating these models with multi-omics analyses (e.g., RNA-seq) of the resultant TME will provide a systems-level understanding of how PKP1 dysregulation alters immune cell composition and function.

In essence, PKP1 functions as a molecular switch at the intersection of tumor cell signaling and immune communication. Its dysregulation, whether through loss or gain, can shape a permissive TME via distinct mechanisms. This emerging paradigm challenges a singular explanation and instead advocates for a nuanced, context-defined understanding of PKP1’s role in cancer immunology. Resolving these mechanisms through targeted experimental approaches will not only clarify fundamental biological processes but also reveal potential therapeutic strategies to modulate the TME by targeting PKP1 or its downstream effectors.

## 4 Potential clinical applications of PKP1

As detailed in preceding sections, PKP1 expression and subcellular localization exhibit remarkable heterogeneity in neoplastic tissues, correlating with tumor type, differentiation status, recurrence, metastasis, and invasive potential. These context-dependent patterns position PKP1 as a promising biomarker with multifaceted clinical utility in tumor differentiation, staging, and prognosis prediction.

### 4.1 Diagnostic value: subcellular localization as a tissue-specific marker

PKP1 exhibits distinct subcellular localization patterns that offer valuable diagnostic insights, particularly in the subtyping of NSCLC. However, consistent with contemporary diagnostic paradigms, the utility of PKP1 is maximized when used as a complementary component within multi-analyte immunohistochemical (IHC) panels rather than as a standalone biomarker. Its membranous expression demonstrates high specificity (97.4%–100%) for distinguishing SCC from adenocarcinoma (Gómez-Morales et al., 2013; Galindo et al., 2020), thereby serving as a reliable adjunct to established markers such as TTF-1 (positive in adenocarcinoma) and p63 (positive in SCC) (Sharma et al., 2022). When used in conjunction with cytokeratin 5/6 (CK5/6) and p63, this triad achieves a classification accuracy of 96.2% (94.6% for adenocarcinoma, 97.6% for SCC) (Galindo et al., 2020). Furthermore, PKP1 demonstrates potential in the early detection of cancer, as evidenced by an increase in promoter methylation frequency from 12.8% in Barrett’s esophagus to 33.3% in high-grade dysplasia and EAC (p < 0.05), highlighting its value as an epigenetic biomarker for risk stratification (Kaz et al., 2012).

The evolving landscape of cancer diagnostics is increasingly emphasizing the integration of artificial intelligence-assisted and digital pathology platforms, where the role of PKP1 is anticipated to expand. By incorporating PKP1 into algorithm-driven panels that integrate IHC, genomic, and morphologic features (e.g., alongside PD-L1 for immune context, TTF-1/Napsin A for adenocarcinoma, and p63 for SCC), its contribution can be dynamically weighted to enhance diagnostic accuracy. This integrated approach not only refines the subtyping of NSCLC but also supports emerging applications in prognostic stratification and therapy selection, thereby positioning PKP1 as a versatile element in the precision oncology toolkit.

### 4.2 Prognostic significance: linking differentiation and metastasis

Across various cancer types (e.g., cutaneous and oropharyngeal SCCs, BCCs), a consistent prognostic pattern is observed: the retention of membranous PKP1 is associated with well-differentiated histology and a reduced risk of metastasis. Conversely, the loss of PKP1 or its cytoplasmic localization is indicative of tumor dedifferentiation, increased invasiveness, and poorer patient outcomes (Papagerakis et al., 2003; Moll et al., 1997). Beyond its individual prognostic significance, PKP1 has been recently recognized as a crucial component of a robust multi-gene prognostic signature for esophageal SCC. The CCND1-PKP1-JUP-ANKRD12 model effectively stratifies esophageal SCC patients into distinct risk categories, independent of other clinicopathological factors. The prognostic value of PKP1 within this signature has been further validated at the protein level, underscoring its clinical importance (Zhang X. et al., 2025).

### 4.3 Therapeutic targeting: disrupting oncogenic networks

**FIGURE 2:**
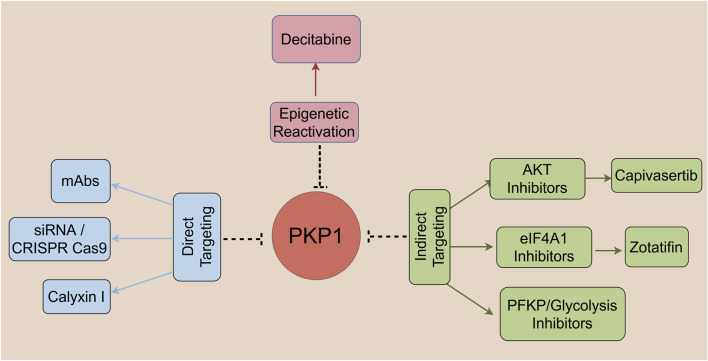
Therapeutic targeting strategies for PKP1 in cancer. Schematic overview of current and potential approaches to target PKP1 or its associated oncogenic networks. Strategies are categorized into three main classes: Direct Targeting (left): Includes monoclonal antibodies (mAbs) and genetic tools (e.g., siRNA/CRISPR-Cas9) aimed at suppressing PKP1 expression or function, as well as natural compounds such as calyxin I identified as high-affinity PKP1 binders. Indirect Targeting (right): Focuses on inhibiting key downstream effectors of PKP1-driven pathways, including AKT inhibitors (e.g., capivasertib), eIF4A1 inhibitors (e.g., zotatifin), and PFKP/glycolysis inhibitors to disrupt metabolic reprogramming. Epigenetic Reactivation (top): Uses demethylating agents such as decitabine to restore PKP1 expression in contexts where its promoter hypermethylation contributes to tumor suppression loss.

PKP1’s involvement in feedforward loops with oncogenic pathways presents potential therapeutic opportunities. It activates MYC to recruit eIF4A1, while MYC transcriptionally upregulates PKP1 via promoter binding (Martin-Padron et al., 2020; Boyero et al., 2022). Furthermore, PKP1 sustains PI3K/AKT signaling through mutual reinforcement with AKT (Moll et al., 1997). Primary strategies targeting PKP1-associated networks include (Figure 2):

Direct PKP1 inhibition: Genetic silencing (siRNA/CRISPR) or monoclonal antibodies disrupting PKP1-MYC interactions (Martin-Padron et al., 2020; Boyero et al., 2022).

Complementary to these strategies, natural compounds, particularly flavonoids, have been identified as potential small-molecule inhibitors of PKP1. An *in silico* study has highlighted calyxins I, a flavonoid, as a high-affinity binder to PKP1, demonstrating superior binding energy compared to the conventional drug afatinib (Pandey et al., 2022). This finding suggests a promising direction for the development of plant-based therapies targeting PKP1, potentially offering reduced side effects.

Downstream pathway blockade: AKT inhibitors (e.g., capivasertib, NCT04305496) (Hu et al., 2025; Turner et al., 2023) or eIF4A1 inhibitors (e.g., zotatifin) (Kuzuoglu-Ozturk et al., 2025; Barranco, 2025) to disrupt oncogenic cascades.

Epigenetic reactivation: Demethylating agents (decitabine, 5-aza-dC) (Carnie et al., 2024) to restore PKP1’s tumor-suppressive functions.

Metabolic targeting: PFKP inhibitors (Ishaq et al., 2024) or glycolytic regulators (Zhang Y. et al., 2025) to disrupt the PKP1-PFKP axis to inhibit glycolysis in PKP1-high tumors (Ritoré-Salazar et al., 2025).

Emerging evidence also indicates a correlation between PKP1 expression and therapeutic response. In esophageal SCC cell lines, the expression of PKP1 and its associated gene signatures have been significantly correlated with sensitivity to various anti-cancer drugs, suggesting that PKP1 may serve as a potential predictive biomarker for chemotherapy response (Zhang X. et al., 2025).

Despite these promising prospects, considerable challenges persist in therapeutically targeting PKP1. A major concern is the potential for on-target toxicity, given the critical role of PKP1 in maintaining epithelial integrity in normal tissues, as demonstrated by the skin fragility phenotype observed in individuals with germline PKP1 deficiency (McGrath et al., 1997). Consequently, future therapeutic strategies must focus on achieving tumor-selective targeting. This could be accomplished by leveraging synthetic lethality interactions specific to PKP1-overexpressing cancer cells or by developing approaches that selectively degrade the oncogenic cytoplasmic/nuclear pools of PKP1 without disrupting its tumor-suppressive membrane-associated functions. Furthermore, nanocarrier-based delivery systems could improve the tumor-specific bioavailability of PKP1-targeted agents.

In conclusion, the context-dependent roles of PKP1 as an oncoprotein or tumor suppressor render it a versatile clinical target. However, the clinical translation of these strategies is challenging due to the dualistic nature of PKP1, necessitating meticulous patient stratification based on PKP1 expression levels and subcellular localization. The integration of multi-omics approaches with preclinical models (e.g., conditional knockout mice), alongside biomarker-driven clinical trials, will be essential for translating the biological insights of PKP1 into effective diagnostics, prognostics, and therapeutic strategies.

## 5 Conclusion and future perspectives

In summary, this review highlights the context-dependent duality of PKP1 in cancer, primarily influenced by its subcellular localization and post-translational modifications. Its role as a critical modulator of adhesion, translation, and signaling pathways underpins its potential clinical utility as a diagnostic and prognostic biomarker across various malignancies. To effectively translate these mechanistic insights into clinical practice, several key challenges must be addressed. For instance, how do signaling pathways (e.g., Wnt/β-catenin and Ca^2+^) dynamically regulate the subcellular shuttling of PKP1 during tumor progression? To comprehensively understand the transitions in models such as Barrett’s esophagus, longitudinal single-cell tracking studies are essential (Kaz et al., 2012). The factors that determine the functional dominance of competing phosphorylation events (e.g., AKT2 vs. RIPK4) remain unclear. Investigating the crosstalk between insulin/IGF1 signaling and differentiation cues may elucidate this antagonism (Wolf et al., 2013; Lee et al., 2017; Fortugno et al., 2022). Furthermore, the role of PKP1 in tumor immune evasion requires further exploration. Understanding its involvement in the activation of MDSC (Huang et al., 2022) and cytokine-mediated immunosuppression (Kim et al., 2023) could provide valuable insights for the development of immunotherapy strategies.

To address the context-dependent duality of PKP1, several research directions should be prioritized. These include the use of advanced preclinical models, such as conditional knockout mice, to investigate the stage-specific functions of PKP1 in tumor initiation and metastasis; the application of multi-omics approaches, particularly single-cell sequencing, to analyze the spatiotemporal dynamics of PKP1 expression and modification; the validation of its clinical utility through prospective trials, such as those for NSCLC subtyping; and the development of targeted strategies, including AKT/PI3K inhibitors or demethylating agents, to modulate PKP1-associated pathways.

In conclusion, the translation of mechanistic insights pertaining to PKP1 into clinical practice necessitates coordinated efforts to connect molecular discoveries with concrete patient outcomes. By overcoming these challenges, PKP1 has the potential to establish itself as a pivotal element within the precision oncology framework, offering integrated diagnostic, prognostic, and therapeutic benefits across various cancer types.

## Abbreviations

ARD, armadillo repeat domain; CTCs, circulating tumor cells; eIF4A1, eukaryotic initiation factor 4A1; EMT, epithelial‒mesenchymal transition; NEs, normal epithelial cells; NPC, nasopharyngeal carcinoma; NSCLC, non-small cell lung cancer; NUPR1, nuclear protein 1; OS, overall survival; PCad, prostate adenocarcinoma; PFS, progression-free survival; PKP1, plakophilin 1; RIPK4, receptor-interacting serine/threonine-protein kinase 4; SCC, squamous cell carcinoma; SILs, squamous intraepithelial lesions; TME, tumor microenvironment.
